# Application of Indocyanine Green Videoangiography in Aneurysm Surgery: Evidence, Techniques, Practical Tips

**DOI:** 10.3389/fsurg.2019.00034

**Published:** 2019-06-20

**Authors:** Pedro Norat, Sauson Soldozy, Mazin Elsarrag, Jennifer Sokolowski, Kaan Yaǧmurlu, Min S. Park, Petr Tvrdik, M. Yashar S. Kalani

**Affiliations:** Department of Neurological Surgery, University of Virginia Health System, Charlottesville, VA, United States

**Keywords:** cerebral aneurysm, indocyanin green, digital subtraction angiogram, surgical microscope, near infra-red

## Abstract

Establishing blood vessel patency in neurovascular surgery is an essential component in treating cerebrovascular disorders. Given the difficulty in confirming complete obliteration of the aneurysm sac, ICG videoangiography has emerged as an intraoperative tool that provides neurosurgeons immediate feedback on the status of vessel flow, allowing for surgical modifications to be made without delay. ICG initially emerged as a tool for assessing hepatic, cardiac, and retinovascular function. It is an inert compound with a high affinity for plasma proteins and fluorescence properties making it the ideal candidate for assessment of vessel patency in neurovascular procedures. Requiring only a bolus peripheral vein injection and integration of a near-infrared imaging device into the surgical microscope, ICG can be visualized without disrupting operating room workflow or the surgical field. Quick response time, high-spatial resolution, and low complication rates are features of ICG videoangiography that prove advantageous when compared to the gold standard intra- and postoperative digital subtraction angiography (DSA). Despite this, ICG is not without limitations, specifically in the setting of atherosclerotic vessels, giant, and complex aneurysms. Additionally, there are instances where DSA may prove superior in detecting vessel stenosis and outflow obstruction, prompting the recommendation of ICG as an adjunct to, rather than complete replacement of DSA. In this article, the authors provide a brief overview of the biochemical properties and historical origins of ICG viedoangiography in addition to discussing its current application in aneurysm surgery.

## Introduction

Indocyanine green (ICG) is a fluorescent compound that has been utilized for decades in a variety of medical applications. Originally approved by the Food and Drug Administration in 1956, ICG was initially used to evaluate cardiac and hepatic function and later gained extensive use in ophthalmology where it was used to enable angiography (1, 2). More recently, it has been used to enhance tissue visualization in oncology, plastic surgery, neurosurgery, and other fields (3).

Given that the physical properties of ICG may be utilized to facilitate vascular imaging, the utility of ICG angiography in cerebrovascular surgery has become quite apparent.

The first description of ICG angiography for aneurysm surgery was in 2003, where Raabe et al. assessed the feasibility and quality of ICG videoangiography in assessing patency of arterial and venous vessels and exclusion of aneurysm sacs (4). In their study, the ability to visually inspect vessel patency with intraoperative ICG angiography allowed for real-time surgical changes that significantly altered the surgical course in a number of cases. Given the importance of maintaining tissue perfusion in neurovascular surgery and the difficulty associated with ensuring complete obliteration of an aneurysm, a number of studies have since explored and championed the utility of ICG angiography in aneurysm surgery (2, 5–12) as well as in other neurosurgical settings, such as resection of AVMs and AVFs (13–15), vascular bypass surgery (16), and tumor resection (17). With regards to aneurysm surgery, ICG angiography is often contrasted with other intraoperative monitoring techniques such as digital subtraction angiography (DSA) (5), intraoperative computed tomography (6), and microvascular doppler sonography (7), with these studies concluding that ICG angiography should complement rather than replace these alternative imaging methods. In this review, the authors discuss the biochemical properties of ICG dye, provide a brief history of its use in the medical setting, as well as discuss methodology and current applications in aneurysm surgery while considering both the advantages and limitations of this technique.

## Biochemical Profile and Properties

**Figure 1:**
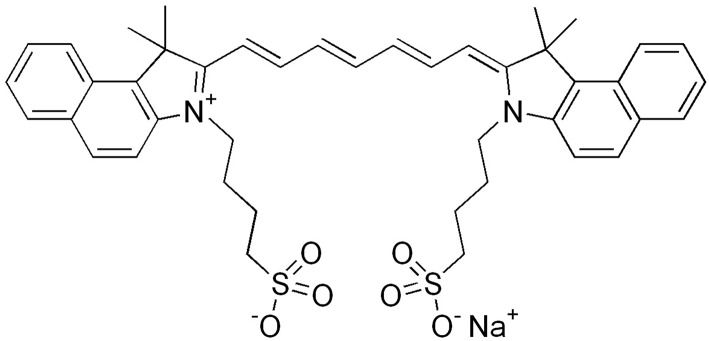
Chemical structure of indocyanine green. Reproduced from File:Indocyanine green.png (18).

Indocyanine green (Figure 1) is a water-soluble, odorless or near odorless tricarbocyanine dye with a strong affinity for plasma proteins (primarily albumin and α- and β-lipoproteins) (1, 19, 20). Once administered, ICG remains 98% bound to plasma proteins; this intravascular sequestration of the dye allows blood vessels to be easily visualized using fluorescence imaging techniques. ICG molecules have a spectral absorption range of 750–800 nm, with a peak emission occurring at approximately 832 nm (1, 3, 21). The range of light wavelengths where biological tissue absorption and scattering are minimum is between 650 and 900 nm, otherwise known as the biological “optical window” (3, 22). As a result, light has maximal tissue penetration at this range of wavelengths. As both the excitation and fluorescence wavelengths of ICG lie within the tissue optical window, the dye may be seen beneath several layers of tissue (3, 21). Furthermore, due to minimal light absorption at this wavelength, tissues have a low degree of near infra-red (NIR) autofluorescence in comparison with ICG, resulting in stark contrast of the dye.

ICG is a relatively safe, nontoxic compound with a lethal dosage (LD_50_) of 50–80 mg/kg. With a typical ICG angiography dose of 0.2–0.5 mg/kg (21), adverse events are seen in <0.1% of patients (primarily hypotension, tachycardia, and urticarial reactions in iodide allergic patients). The dye is quickly removed from circulation by the liver, mostly clearing within 10–20 min (1, 2). Experiments suggest that ICG is excreted exclusively by the liver into bile with no known metabolites (23).

## Historical Context

Originally introduced for NIR photography by Kodak research laboratories in 1955, ICG was approved for clinical use by the FDA just a year later in 1956 (23). ICG was initially utilized for quantitative measurements in assessing liver function, in the field of cardiology for determining cardiac output in valvular and septal defects (24), and eventually in the field of ophthalmology beginning in the 1970s (3). The first application of ICG in cerebrovascular angiography was experimentally performed on exposed dog brain, reported by Kogure et al. (25). In this study, a wide parietotemporal craniotomy was performed with ICG dye being injected into the left thyroid artery allowing for gross visualization of the middle cerebral artery (MCA) distribution. The authors heralded the “simplicity of technique and employment of standard equipment” noting “it is entirely feasible that a photographic setup such as described in this article could be easily moved into the neurosurgical operating suite.” Their prediction would reign true and was made even more feasible given the technological advancements that occurred in the following decades since their experiment was performed. This includes visualizing ICG fluorescence through a surgical microscope as described by Kuroiwa et al. (26), where they reported clear visualization of human brain surface vessels through the dura mater after intravenous injection of ICG.

**Figure 2:**
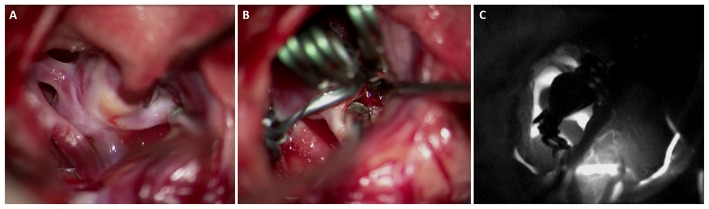
Middle cerebral artery aneurysm with the neck dissected and clear view of the parent vessel, and its two branches **(A)**, after retraction showing the neck of the clipped MCA aneurysm **(B)**, and intraoperative ICG videoangiography showing the patency of the branches of the MCA and complete exclusion of the aneurysm from the circulation **(C)**.

The first description of NIR videoangiography using ICG dye for intraoperative visualization in aneurysm surgery was described in 2003. Raabe et al. (4) noted that in three cases, the ICG angiographic findings significantly changed the course of the surgical procedure, thereby suggesting that this technique may be implemented as an adjunct in neurovascular procedures (Figure 2). In addition, the ICG angiography results were noted to be comparable to DSA in elucidating patency of all vessel types, including small and perforating arteries (<0.05 mm). The integration of NIR technology into the operating microscope would set the stage for ICG videoangiography in neurovascular surgery in the twenty-first century.

## Technique

### Dosage

The recommended dose of ICG injected peripherally for angiography is between 0.2 and 0.5 mg/kg, not to exceed a maximum dose of 5 mg/kg. The standard dose is injected into a peripheral vein as a bolus of 25 mg dissolved in 5 ml of water (2, 3). Within 1–2 s following injection, the ICG dye binds to plasma proteins and has a plasma half-life of 3–4 min. After 10 min, a repeat dose can be administered or until after the ICG dye clears (2). Caloric restriction has been shown to increase plasma clearance at doses below 0.5 mg/kg (27).

### Microscopy

The technical integration of ICG videoangiography into the optical path of the surgical microscope was first described by Raabe et al. (21) and implemented by Carl Zeiss Co. (Oberkochen, Germany). Given that its fluorescent wavelength is in the near-infrared (NIR) spectrum (700–2,500 nm), the dye cannot be seen by the naked eye, and a NIR imaging device must be available to allow vessel visualization. A simple ICG device consists of a NIR light source, an NIR sensitive sensor, and filters used to block light at other wavelengths (3, 21). This allows users to obtain high-resolution NIR images without ambient and excitation light interference. A light source illuminating the operative field has a wavelength comprising the ICG absorption band (range 700–850 nm, peak 805 nm). The setup entails a uniquely designed dielectric filter that allows passage of light in the NIR wavelength that fits the exact absorption band of ICG. An integrated beam splitter in the microscope directs the ICG fluorescence light (range 780–950 nm, peak 835 nm) toward a black-and-white camera, with an observation band-pass filter being used to detect the ICG fluorescence (21).

## Current Applications

The neurosurgeon's goal for aneurysm surgery is complete exclusion of the aneurysm from the circulation while preserving the parent, perforating, and branching vessels. To achieve this, many intraoperative monitoring techniques, such as microvascular Doppler, monitoring of somatosensory and motor evoked potentials, DSA, and more recently ICG videoangiography, may be utilized. Since the publication of Raabe et al. (4), ICG has been greatly studied and many publications have shown its importance in the microsurgical management of aneurysms; consequently, ICG has become a widely commonplace tool for identification of mis-clipping in aneurysm surgery despite DSA being considered the gold standard technique for intraoperative monitoring in aneurysmal clipping (2, 11, 21, 28, 29). However, intraoperative DSA may be challenging to implement in many centers worldwide due to its requirement of large logistical support, high cost burden, prolonged time frame, and invasive nature. In contrast, ICG is noninvasive with a lower cost burden and quicker image acquisition.

In a prospective two-center trial published in 2005, Raabe et al. (2) directly compared surgical microscope-based ICG videoangiography with intra- or postoperative DSA after aneurysm clipping. The authors described several advantages, including a lower complication rate (0.1% vs. 0.4–2.6%), a faster time frame of 2 min, and higher-spatial resolution that allows for observation of small perforators or cortical arteries of submillimeter diameter that cannot be assessed with intraoperative DSA. ICG angiography was reported to be limited in the setting of atherosclerotic or calcified vessels, thrombosed aneurysms, and giant or complex aneurysms; although, ICG disclosed remnant aneurysms mainly in atherosclerotic aneurysms (*p* < 0.05) (30). In cases where ICG angiography missed stenotic vessels, they were found to be likely hemodynamically irrelevant as seen on postoperative DSA. Overall, a correlation of 90% was reported between ICG and DSA, with the authors reporting no cases where inconsistent findings on postoperative DSA lead to reopening and repositioning of the clip (2).

**Figure 3:**
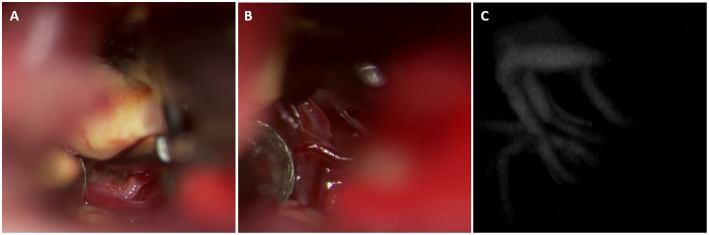
This basilar tip aneurysm was exposed using a modified orbitozygomatic approach. The perforators arising from the posterior aspect of the basilar apex and P1 can be visualized **(A)**. These perforators are dissected from the aneurysm dome and excluded from the clip construct **(B)**. Post clip ICG demonstrates preservation of the perforators **(C)**.

One major utility of ICG is its ability to confirm microsurgical anatomy in a variety of aneurysm cases. de Oliveira et al. (31) have described their experience with 93 ICG videoangiography studies for monitoring the flow in perforating arteries (Figure 3) during intracranial aneurysm surgery. They concluded that ICG videoangiography was able to visualize, during the surgical procedure, the small perforating arteries that cannot be detected by intraoperative DSA (31). In addition, Nagai et al. (32) reported a rare case of a ruptured dissecting aneurysm in the A1 segment. Using ICG, the lateral and medial groups of the basal perforating arteries of the anterior communicating artery complex of the ACA were identified prior to clipping, thereby minimizing the risk of complications related to occluding perforator vessels. Other series have been published that further corroborate these findings (8–14, 16, 17, 28, 33–35).

## Advantages and Limitations

In concordance with the benefits stated earlier, Sharma et al. (11) demonstrated in 112 patients an 8% clip repositioning rate during surgery after the use of ICG videoangiography, elucidating the clear advantage of having ICG available for use. Despite this, a disagreement between ICG and DSA was observed in 4% of the cases (5 cases−2 patients with anterior communicating aneurysms and ophthalmic ICA aneurysms and 1 middle cerebral artery bifurcation), with discordance being significant higher in ophthalmic ICA when compared with other aneurysm locations (*p* = 0.04; OR = 10.8). Dashti et al. (36) in their series of 239 patients found the rate of unexpected neck residuals to be about 6%; however, neck residuals were significantly more frequent in deep-sited aneurysms (64%). They ultimately concluded that ICG videoangiography is a reliable intraoperative tool for aneurysm surgery, with the exception of giant, complex, and deep-sited aneurysms.

Limitations of ICG videoangiography include trouble visualizing aneurysm residuals after clipping when compared with DSA, inability to visualize areas outside the field of the microscope, and difficulty visualizing of arteries at the depth of the surgical field and residual neck in calcified and thrombosed aneurysms. While Raabe et al. (2) report ICG videoangiography can be used as an independent form of angiography, the majority of other studies recommend this technique as an alternative to or as complementary to intraoperative DSA during aneurysm surgery. In fact, a recent systematic review meta-analysis concluded that ICG videoangiography should serve only a complementary role to DSA rather than fully replace it given a 6.1% rate of mis-clippings not detected by ICG angiography among 1,465 clipped aneurysms as compared to a 4.5% rate of mis-clippings not detected by DSA among 849 clipped aneurysms (5). While not as precise as DSA, the greatest advantage of ICG videoangiography is that it provides real-time information regarding aneurysmal exclusion from the circulation, as well as patency of parent and branch vessels. If necessary, clip repositioning can be performed within minutes given the immediate visual feedback of ICG. Additionally, ICG is susceptible to false negatives further pointing toward its role as an adjunct to DSA (37–39).

## Future Directions

Given the advantages and ease of use of ICG videoangiography, the dearth of studies examining its utility in other neurovascular procedures suggests that it may be underutilized for these procedures. Examples of these surgeries include ablation of arteriovenous malformations and fistulas along with manipulation and resection of highly vascularized tumors and lesions. The scarcity of data examining ICG videoangiography for these procedures may be a result of their infrequent incidence. Nonetheless, reports on the nuances of employing ICG videoangiography in these procedures may help to further define its specific roles and situational uses. We encourage groups to publish their techniques and experiences with ICG videoangiography in these areas.

One limitation of ICG videaoangiography is its inability to provide information on unexposed vessels. In order to bypass this limitation, several groups have interfaced the indocyanine NIR imaging technology to neuroendoscopes (8, 40–43). The ICG videoangiography-neuroendoscope may extend the benefits of ICG videoangiography to deep seated lesions and other difficult to visualize vessels without expanding the dissection. Cho et al. (41) report higher detection rates of branch orifices and more exact clip position with the ICG videoangiography-neurendoscope compared with commercial microscopic ICG videoangiography. Another mechanism by which the line of sight limitation has been addressed is with integration of ICG angiography and robotic surgery. The union of ICG angiography with robotic surgery has proven to be valuable in other disciplines such as surgical oncology, (44) demonstrating that this technology is feasible.

In addition to extending ICG videoangiography to lesions and vessels not readily exposed, the robotic system has other benefits which could improve aneurysm surgery. The computer of the robotic console allows additional information to be integrated and simultaneously displayed intraoperatively (44), and the increased precision of movements and ability to navigate narrow anatomical cavities may allow access to deep seated lesions while minimizing brain retraction and dissection. Thus, the expansion of robotics into neurosurgery may be a novel method to incorporate NIR imaging technology and use ICG videoangiography to examine vasculature that is not immediately exposed.

A known drawback to ICG videoangiography is its poor ability to visualize thrombosed, calcified, and buried vessels and aneurysms. This may be in part due to the relatively modest tissue penetration by light at these wavelengths in comparison to other wavelengths. Contrast between ICG and surrounding tissue indeed stems from the fluorescent wavelengths of ICG lying within the tissue optical window. However, Smith et al. (45) discuss the existence of a second optical window, with the first window spanning from 650 to 950 nm and the second from 1,000–1,350 nm. They further state that the first window may not be optimal due to tissue autofluorescence producing substantial background noise and the tissue penetration depth being limited to between 1 and 2 cm. Indocyanine green fluorescing in this first window may significantly detract from the visual quality of ICG angiography. Image acuity may therefore be optimized by utilizing compounds that fluoresce in second optical window. Smith et al. (45) cite simulations and modeling studies suggesting that improvement of signal-to-noise ratios by over 100-fold is possible by using quantum dot fluorophores that emit light at 1,320 nm as opposed to 850 nm. However, currently tested compounds that fluoresce at this range are limited due to safety and toxicity as well as technical challenges associated with imaging technology. Nonetheless, the development of both effective and biologically compatible fluorescent substances that operate within the second optical window may have the potential to greatly enhance fluorescent imaging.

Many of the advantages of ICGVA derive from its safety, convenience and cost effectiveness; preservation of these qualities is necessary for a contrast agent to function optimally. Currently, ICG is the only FDA approved near-infrared dye (3, 22). Investigators have explored both structural analogs of indocyanine as well as novel NIR contrast agents (3). Other areas of improvement include enhancements in NIR imaging technology to capture the broad fluorescence peak of ICG while also effectively filtering out background noise. Lastly, technical developments in flow dynamics analysis may improve visualization after multiple indocyanine loads, since rapid ICG reinjections generally suffer from lower contrast due to residual ICG inside the vessels (3). In addition, the versatility of ICG allows it to be paired with multiple intraoperative monitoring techniques such as somatosensory evoked potential (SSEP) and motor evoked potential (MEP) (46). Overall, there is much that remains to be explored in the field of fluorescent angiography and refinements in technology are inevitable.

## Conclusion

In this article, the authors review the mechanism of action of ICG angiography and examine the evolution of its applications over time, nuances in technique, and clinical implementation. The reviewed data demonstrates that ICG angiography is indeed quite helpful in vascular mapping for cerebral aneurysm surgery and may certainly alter intraoperative decision making. However, its exact role depends on anatomic features of the particular lesion being treated and the technology is ultimately limited by lower accuracy compared to the gold-standard of digital subtraction angiography. Based on these findings, the highest degree of imaging accuracy may be achieved by employing multiple imaging technologies, and we encourage surgeons to use multimodal imaging when possible during aneurysm clipping surgery.

## Author Contributions

MK, PN, and SS: conception and design. PN, SS, and ME: acquisition of data and drafting the article. All authors critically revising the article. All authors reviewed submitted version of manuscript. MK approved the final version of the manuscript on behalf of all authors.

### Conflict of Interest Statement

The authors declare that the research was conducted in the absence of any commercial or financial relationships that could be construed as a potential conflict of interest.
